# Relationship between women’s decision-making power over their own health care and use of modern contraception in the Democratic Republic of the Congo: a cross-sectional secondary data analysis

**DOI:** 10.1186/s12905-021-01450-x

**Published:** 2021-08-21

**Authors:** Megan G. Butler, Melanie Walker, Lesley A. Pablo, Susan A. Bartels

**Affiliations:** 1 Department of Biomedical and Molecular Sciences, Queen’s University, Kingston, ON K7L 3N6 Canada; 2 Department of Emergency Medicine, Queen’s University, Kingston, ON K7L 2V7 Canada; 3 Department of Public Health Sciences, Queen’s University, Kingston, ON K7L 3N6 Canada

**Keywords:** Decision-making, Democratic Republic of the Congo, Demographic and health survey, Health care, Modern contraception, Reproductive health

## Abstract

**Background:**

In sub-Saharan Africa, the use of modern contraception (MC) is a critical intervention aimed at reducing mortality rates associated with unintended, high-risk pregnancies. However, among Congolese women aged 15–49, the prevalence of MC use is low. Research suggests that women’s general participation in decision-making is important in increasing MC use. However, little is known about the specific role of women’s decision-making power over their own health care and how it relates to MC use. Thus, this study aimed to investigate the relationship between women’s decision-making power over their own health care and use of MC.

**Methods:**

A cross-sectional secondary data analysis was conducted using the most recent data from the 2013–2014 Democratic Republic of the Congo (DRC) Demographic and Health Survey. Women who were considered in need of contraception based on their family planning preferences were included in the study population (N = 6422). Multivariate logistic regression was used to determine whether women’s decision-making power over their own health care was associated with the use of MC.

**Results:**

Only one in ten women reported using a modern method of contraception. Logistic regression showed that women who made decisions alone regarding their own health care were more likely to use MC than women who had no say in these decisions, even after controlling for important covariates (OR 1.48; 95% CI 1.00, 2.17).

**Conclusion:**

The results of this study lend further support that promoting women’s autonomy and right to independently make decisions regarding their own health may be important in increasing the use of MC in the DRC. However, in order to effectively empower women to negotiate for the use of MC, qualitative research is needed to further assess the relationship between decision-making power and MC use.

## Background

In sub-Saharan Africa, the use of modern contraception (MC) is a critical part of many maternal health interventions that aim to reduce maternal mortality rates associated with unintended, high-risk pregnancies [1]. Additionally, using MC to increase the length of interpregnancy intervals can improve perinatal health outcomes by reducing the risk of low birthweight and premature birth [1]. However, as of 2013, only eight percent of non-pregnant women aged 15–49 in the Democratic Republic of the Congo (DRC) reported using MC [2]. This represents a less than two percent increase from 2007 [3]. Furthermore, this is very low, even in comparison to other countries in sub-Saharan Africa such as Ghana and Kenya, where prevalence rates of MC use are 22% and 53%, respectively [4, 5]. The National Strategic Plan for Family Planning in DRC aims to increase the prevalence of MC use to 19.0% by 2020 [6]. Progress towards this goal has been made, particularly in the capital city, Kinshasa, where the prevalence of MC use among women aged 15–49 has increased from 18.5% in 2013 to 26.7% in 2017 [7, 8]. In the conflict-affected eastern regions of the DRC, low prevalence of MC use has been attributed to a lack of availability of family planning methods [9]. Previous studies have shown that MC tends to be utilized by women in conflict-affected areas of the DRC when it is available to them [9, 10]. Additionally, there are a variety of sociocultural barriers to accessing contraception in the DRC, including religious beliefs that God should determine the number of children that a woman bears [11, 12]. Previous studies have also found that factors such as wealth, education level, and urban–rural residence are important determinants of MC use [13–15]. That said, further investigation into the reasons for the current climate of contraceptive use in the DRC is warranted.

Women’s general participation in decision-making has been found to be an important factor in increasing the use of MC [13, 15, 17]. However, these studies measured women’s decision-making using a composite variable [13, 15, 17]. Therefore, the specific relationship between women’s decision-making power over their own healthcare and the use of MC has been minimally explored. Two studies in Nigeria found that women who have a say in decisions regarding their own health care are more likely to use MC than women who have no say [14, 18]. Interestingly, a study of young women in Bangladesh found that women who make health care decisions jointly with their partner are more likely to use MC than women who make health care decisions alone [19]. Furthermore, in the DRC, women who report using a modern method of contraception commonly state that support from their partner was a key determinant in their use [11, 12]. These findings support the model of relational autonomy, which emphasizes the importance of having social supports in decisions regarding reproductive health care [20].

Therefore, though women’s participation in reproductive health care decisions has been observed in a few studies to be important in MC use, it is unclear whether autonomous decision-making versus joint decision-making regarding women’s health care is more strongly associated with MC use [20]. Additionally, whether or not a woman participates jointly in decisions about MC may have implications for the type of MC used [21].

Previous literature has investigated the association between decision-making and the use of MC [13–15, 17–19, 21]. However, no study in the DRC has compared the relationship between women having independent, joint or no say over their own health care and current use of MC. Further research is needed to better understand the role of health care decision-making power in an effort to contribute to the reproductive health of women in the DRC. Thus, our primary objective was to investigate the relationship between Congolese women’s decision-making power over their own health care and current use of MC. It was hypothesized that women’s participation, and in particular joint participation with their partner, in decisions about their health care would be associated with an increased likelihood of using MC. Furthermore, among current users of MC, our secondary objective was to examine the association between women’s decision-making power over their own health care and the method of MC used.

## Methods

### Study setting and participants

This cross-sectional study was based on data from the 2013–2014 Democratic Republic of the Congo Demographic and Health Survey (DRC-DHS II). The DRC-DHS is a household survey conducted periodically at the population level using a multi-stage stratified cluster sampling technique [2]. Participation in the survey was voluntary and informed consent was obtained from all participants [2]. The surveys include modules on a variety of topics such as fertility, family planning, maternal and child health, and HIV/AIDS [2, 22]. Additionally, the surveys contain an array of questions related to demographic and sociocultural factors [2]. This study uses variables from the women’s questionnaire and household questionnaire [2]. Further information on data collection and the questionnaires used is available elsewhere [2].

**Fig. 1:**
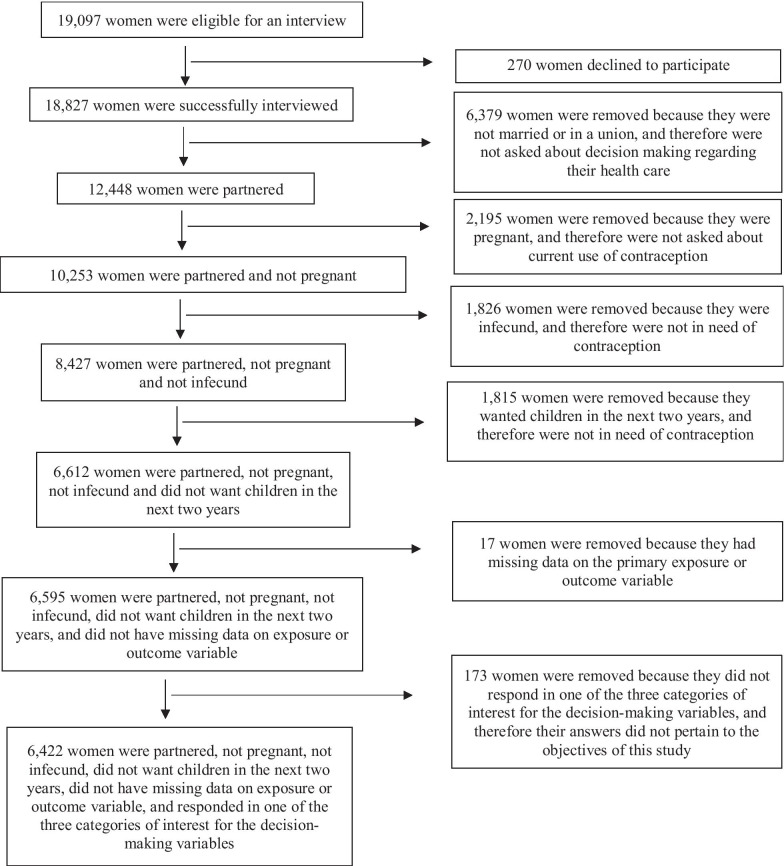
From the 18,827 women successfully interviewed, 6,422 remained in the final study population

Overall, a total of 18,171 households were surveyed, and 19,097 women between the ages of 15 and 49 were asked to participate [2]. Of these women, 18,827 were successfully interviewed and therefore included in the initial study population [2]. For this study, women were then excluded if they were unpartnered, were pregnant, were infecund, desired children in the next two years, had missing data on the exposure or outcome variable or did not respond in one of the three categories of interest for the decision-making variables. Overall, 6422 women were considered in need of contraception based on their family planning preferences and therefore were included in the analysis (Fig. 1).

### Measures

The primary exposure variable in this study was women’s decision-making power over their own health care. This was assessed with the question: “Who usually makes decisions about health care for yourself?” [2]. The possible responses were: (*a*) respondent, (*b*) husband/partner, (*c*) respondent and husband/partner jointly, (*d*) someone else, (*e*) other [2]. Only individuals that responded in the first three categories were included in the analysis. This was because the ‘someone else’ and ‘other’ categories could not be meaningfully interpreted for the purposes of this study as it was unclear who the decision maker(s) were. Additionally, only a small number of women responded in these categories.

The primary outcome of interest was the current use of a modern method of contraception. During the survey, women were asked if they or their partner were currently using any method, or doing anything, to avoid or delay pregnancy [2]. Women who responded positively were then asked which method they were using [2]. A dichotomous variable was created, and women were categorized as either users or non-users of MC. Non-users of MC included both women who were not using contraception and women who were using non-modern methods, such as periodic abstinence or withdrawal [2]. Women were categorized as users of MC if they reported using any of the following methods: male sterilization, female sterilization, injectable, intrauterine device (IUD), implant, diaphragm, pill, male condom, female condom, emergency contraception, vaginal methods (jelly, foam and spermicide), the cycle necklace (or fixed day method) or other modern method [2]. The category of “other modern method” was created by the DHS and was not defined in the DRC-DHS II report.

Other independent variables were also considered based on their roles as confounders in related literature [13–15, 17–19]. Socio-demographic variables included women’s current age, level of education, employment status, age at first cohabitation, parity, urban–rural residence, province and religion. Women’s current age was grouped into 5-year age groups in accordance with related literature [23, 24]. Additionally, socioeconomic status was analyzed with the use of a household wealth index [22, 25]. Finally, variables pertaining to women’s decision-making in other domains were also considered as potential confounders [18, 19]. These included decisions about large household purchases, visits to family or relatives, the use of the respondent’s earnings and the use of the partner’s earnings. Consistent with the primary exposure variable, only individuals who responded in one of the three categories of interest were retained in the analysis.

### Statistical analysis

Descriptive statistics, including means and frequencies, were calculated to describe the characteristics of the study population. Differences in the prevalence of MC use across demographic characteristics of interest were evaluated using t-tests or chi-square tests as appropriate. Bivariate logistic regression was carried out to evaluate the association between the primary exposure variable and, independently, potential covariates and the current use of MC. Covariates significant at *p* < 0.10 were included in the initial multivariate model [26]. A backwards elimination technique was used to obtain the final multivariate model [27]. This involved the stepwise removal of the covariate with the highest *p*-value until the least significant covariate had *p* < 0.20 [27]. Upon removal of a covariate, the model with and without the covariate were compared to assess the change in the odds ratio of the primary exposure variable [27, 28]. A change of more than 10% was considered indicative of confounding and the variable was retained in the final multivariate model [27, 28]. The final multivariate model only contained individuals with complete data on all model variables. All regression analyses controlled for the multi-stage cluster sampling design used in data collection [2].

To check for multicollinearity between the five decision-making variables, linear regression analysis was used to generate collinearity diagnostics [29]. Additionally, the Hosmer–Lemeshow goodness of fit test was conducted for the final multivariate models [30]. SPSS 25.0 was used for all data analyses (SPSS Inc., Armonk, NY). STROBE cross-sectional reporting guidelines were used in the development of this paper [31].

## Results

### Characteristics of the study population

The study population included 6,422 Congolese women aged 15 to 49 years. One in ten women reported currently using MC (Table 1). About half of the women participated in decisions regarding their own health care: 35.9% reported making decisions jointly with their partner and 10.0% reported making decisions independently. One-third of the women lived in an urban region (32.5%) and had secondary or higher level of education (36.9%). Among women who reported using MC, approximately two-thirds lived in an urban region (61.6%) and had a secondary or higher level of education (62.4%). Additionally, two-thirds (63.1%) of the women using MC were from the fourth (25.3%) and fifth (37.8%) highest wealth quintiles. Moreover, 14.5% of the women using MC made health care decisions alone, compared to 9.5% of the women who did not use MC. Finally, more than half of MC users made joint decisions with their partner about their partner’s earnings (56.4%).

**Table 1:** Unweighted distribution of modern contraceptive use by characteristics of the study population

	Currently using modern contraception			
	Total [n(%)] (n = 6422)	Yes [n(%)] (n = 641)	No [n(%)] (n = 5781)	P-value^a^
Decision maker for respondent’s healthcare				< .001
Partner alone	3477 (54.1)	300 (46.8)	3177 (55.0)	
Respondent and partner	2304 (35.9)	248 (38.7)	2056 (35.6)	
Respondent alone	641 (10.0)	93 (14.5)	548 (9.5)	
Decision maker for partner’s earnings				< .001
Partner alone	2838 (44.2)	205 (32.0)	2633 (45.6)	
Respondent and partner	2964 (46.2)	361 (56.4)	2603 (45.0)	
Respondent alone	618 (9.6)	74 (11.6)	544 (9.4)	
Missing	2			
Decision maker for visits to family/relatives				< .001
Partner alone	3035 (47.3)	258 (40.2)	2777 (48.1)	
Respondent and partner	2251 (35.1)	227 (35.4)	2024 (35.0)	
Respondent alone	1134 (17.7)	156 (24.3)	978 (16.9)	
Missing	2			
Decision maker for large household purchases				< .001
Partner alone	2612 (40.7)	208 (32.5)	2404 (41.6)	
Respondent and partner	2792 (43.5)	312 (48.8)	2480 (42.9)	
Respondent alone	1013 (15.8)	120 (18.8)	893 (15.5)	
Missing	5			
Decision maker for respondent’s earnings				< .001
Partner alone	1437 (22.4)	76 (11.9)	1361 (23.6)	
Respondent and partner	1822 (28.4)	200 (31.3)	1622 (28.1)	
Respondent alone	1206 (18.8)	164 (25.7)	1042 (18.1)	
Respondent did not have earnings	1940 (30.3)	199 (31.1)	1741 (30.2)	
Missing	17			
Wealth Quintiles				< .001
Poorest	1573 (24.5)	54 (8.4)	1519 (26.3)	
Poorer	1421 (22.1)	90 (14.0)	1331 (23.0)	
Middle	1308 (20.4)	93 (14.5)	1215 (21.0)	
Richer	1148 (17.9)	162 (25.3)	986 (17.1)	
Richest	972 (15.1)	242 (37.8)	730 (12.6)	
Highest Level of Education				< .001
No education	1285 (20.0)	56 (8.7)	1229 (21.3)	
Primary education	2770 (43.1)	185 (28.9)	2585 (44.7)	
Secondary or higher	2367 (36.9)	400 (62.4)	1967 (34.0)	
Urban–rural residence				< .001
Rural	4334 (67.5)	246 (38.4)	4088 (70.7)	
Urban	2088 (32.5)	395 (61.6)	1693 (29.3)	
Province of Residence				< .001
Kinshasa	443 (6.9)	110 (17.2)	333 (5.8)	
Bandundu	960 (14.9)	85 (13.3)	875 (15.1)	
Bas-Congo	334 (5.2)	77 (12.0)	257 (4.4)	
Equateur	1024 (15.9)	56 (8.7)	968 (16.7)	
Kasai-Occidental	579 (9.0)	45 (7.0)	534 (9.2)	
Kasai-Oriental	715 (11.1)	52 (8.1)	663 (11.5)	
Katanga	713 (11.1)	42 (6.6)	671 (11.6)	
Maniema	300 (4.7)	29 (4.5)	271 (4.7)	
Nord-Kivu	370 (5.8)	63 (9.8)	307 (5.3)	
Orientale	687 (10.7)	57 (8.9)	630 (10.9)	
Sud-Kivu	297 (4.6)	25 (3.9)	272 (4.7)	
Age				.033
15–19	414 (6.4)	31 (4.8)	383 (6.6)	
20–24	1254 (19.5)	118 (18.4)	1136 (19.7)	
25–29	1628 (25.4)	142 (22.2)	1486 (25.7)	
30–34	1231 (19.2)	145 (22.6)	1086 (18.8)	
35–39	1035 (16.1)	106 (16.5)	929 (16.1)	
40–44	637 (9.9)	70 (10.9)	567 (9.8)	
45–49	223 (3.5)	29 (4.5)	194 (3.4)	
Age at first cohabitation^b^				.013
	17.90 (8–44)	18.25 (11–38)	17.86 (8–44)	
Currently Working				.077
Yes	4881 (76.1)	469 (73.3)	4412 (76.4)	
No	1532 (23.9)	171 (26.7)	1361 (23.6)	
Missing	9			
Religious Affiliation				.875
Christian	6142 (95.9)	610 (95.8)	5532 (95.9)	
Non-Christian	264 (4.1)	27 (4.2)	237 (4.1)	
Missing	16			
Number of Children				.364
Less than three	1946 (30.3)	207 (32.3)	1739 (30.1)	
Three to five	2836 (44.2)	267 (41.7)	2569 (44.4)	
More than five	1640 (25.5)	167 (26.1)	1473 (25.5)	

OR is odds ratio; CI is confidence interval

^a^P-values from Pearson’s Chi-Square Tests

^b^Mean and range values are reported for age at first cohabitation. P-values from Pearson’s Chi-Square Tests

### Association between women’s decision-making power over their own health care and current use of modern contraception

Table 2 shows the results of the adjusted logistic regression analysis evaluating the association between women’s decision-making power over their own health care and current use of MC. The final multivariate regression model controlled for decision maker for partner’s earnings, decision maker for visits to family/relatives, wealth quintile, highest level of education, urban–rural residence, province of residence, and age. Results showed that women who made health care decisions alone had higher odds of MC use than women who had no say, even after controlling for potential confounders (OR 1.48; 95% CI 1.00, 2.17) (Table 2). Furthermore, the odds of using MC were 1.44 times greater for women who participated jointly with their partner in decisions about their partner’s earnings than women who had no say (OR 1.44; 95% CI 1.09, 1.89). In addition to decision-making, there were other variables that were significantly associated with the current use of MC. Women from the fourth and fifth highest wealth quintiles had higher odds of MC use [(OR 2.25; 95% CI 1.40, 3.60) and (OR 2.91; 95% CI 1.70, 4.98)] than women from the lowest wealth quintile. Additionally, women in Katanga had lower odds of MC use than women in Kinshasa (OR 0.43; 95% CI 0.27, 0.67). However, women in Bas-Congo and Nord-Kivu had higher odds of MC use [(OR 3.17; 95% CI 1.83, 5.48) and (OR 1.90; 95% CI 1.16, 3.12)]. Furthermore, the odds of using MC were approximately twice as high for women living in urban regions compared to women living in rural regions (OR 1.90; 95% CI 1.30, 2.79). Finally, women with secondary or higher levels of education had higher odds of MC use than women with no education (OR 1.74; 95% CI 1.10, 2.78).

**Table 2:** Odds ratios of current use of modern contraception among Congolese women (n = 6,392)

	Adjusted* OR [95% CI]
Decision maker for respondent’s health care	
Partner alone	1.00
Respondent and partner	1.02 [0.75, 1.39]
Respondent alone	**1.48 [1.00, 2.17]**
Decision maker for partner’s earnings	
Partner alone	1.00
Respondent and partner	**1.44 [1.09, 1.89]**
Respondent alone	0.99 [0.65, 1.50]
Decision maker for visits to family/relatives	
Partner alone	1.00
Respondent and partner	0.86 [0.65, 1.14]
Respondent alone	1.11 [0.79, 1.56]
Wealth Quintiles	
Poorest	1.00
Poorer	1.43 [0.89, 2.29]
Middle	1.28 [0.79, 2.05]
Richer	**2.25 [1.40, 3.60]**
Richest	**2.91 [1.70, 4.98]**
Highest Level of Education	
No education	1.00
Primary education	1.07 [0.69, 1.64]
Secondary or higher	**1.74 [1.10, 2.78]**
Urban–rural residence	
Rural	1.00
Urban	**1.90 [1.30, 2.79]**
Province of Residence	
Kinshasa	1.00
Bandundu	1.39 [0.83, 2.32]
Bas-Congo	**3.17 [1.83, 5.48]**
Equateur	0.94 [0.54, 1.65]
Kasai-Occidental	1.20 [0.55, 2.61]
Kasai-Oriental	0.67 [0.40, 1.12]
Katanga	**0.43 [0.27, 0.67]**
Maniema	1.43 [0.55, 3.72]
Nord-Kivu	**1.90 [1.16, 3.12]**
Orientale	1.05 [0.67, 1.66]
Sud-Kivu	1.04 [0.53, 2.03]
Age	
15–19	1.00
20–24	1.16 [0.65, 2.08]
25–29	0.86 [0.51, 1.46]
30–34	1.41 [0.79, 2.49]
35–39	0.86 [0.49, 1.52]
40–44	0.95 [0.51, 1.76]
45–49	1.04 [0.48, 2.27]

OR is odds ratio; CI is confidence interval; Complex sample plan was used to weight data and control for the sampling method

Bold = significant at p < 0.05

*Model controls for decision maker for partner’s earnings, decision maker for visits to family/relatives, wealth quintile, highest level of education, urban–rural residence, province of residence, and age

### Association between women’s decision-making power over their own health care and method of contraception used

Among current users of MC (n = 612), 297 women reported using a male method and 315 women reported using a female method of contraception. One-third (37.2%) of users of female methods of MC lived in two provinces: Kinshasa (21.3%) and Nord-Kivu (15.9%) (Table 3). Only 6.7% of users of male contraception were between the ages of 40 and 49, while 22.5% of users of female contraception were in this age group. Furthermore, 43.8% of women using male contraception had less than three children in comparison to 21.3% of women using female contraception. For decision making for visits to family/relatives, 47.1% of women using male contrapcetion reported their partner making the decision alone compared to 33.3% of women using female contrapcetion. Finally, 41.9% of users of female contrapcetion were in the richest wealth quintile in comparison to 32.7% of users of male contraception.

**Table 3:** Unweighted descriptive statistics of the method of modern contraception used among current users of modern contraception

	Method of Modern Contraception Used^a^			
	Total (%) (n = 612)	Male Method (%) (n = 297)	Female Method (%) (n = 315)	P-value^b^
Decision maker for respondent’s health care				.216
Partner alone	286 (46.7)	148 (49.8)	138 (43.8)	
Respondent and partner	237 (38.7)	112 (37.7)	125 (39.7)	
Respondent alone	89 (14.5)	37 (12.5)	52 (16.5)	
Decision maker for partner’s earnings				.923
Partner alone	194 (31.8)	93 (31.3)	101 (32.2)	
Respondent and partner	346 (56.6)	168 (56.6)	178 (56.7)	
Respondent alone	71 (11.6)	36 (12.1)	35 (11.1)	
Missing	1			
Decision maker for visits to family/relatives				.002
Partner alone	245 (40.0)	140 (47.1)	105 (33.3)	
Respondent and partner	216 (35.3)	93 (31.3)	123 (39.0)	
Respondent alone	151 (24.7)	64 (21.5)	87 (27.6)	
Decision maker for large household purchases				.052
Partner alone	196 (32.1)	108 (36.8)	88 (28.0)	
Respondent and partner	300 (49.1)	132 (44.2)	168 (53.5)	
Respondent alone	115 (18.8)	57 (19.0)	58 (18.5)	
Missing	1			
Decision maker for respondent’s earnings				.964
Partner alone	72 (11.8)	37 (12.5)	35 (11.1)	
Respondent and partner	192 (31.5)	92 (31.1)	100 (31.8)	
Respondent alone	156 (25.6)	75 (25.3)	81 (25.8)	
Respondent did not have earnings	190 (31.1)	92 (31.1)	98 (31.2)	
Missing	2			
Wealth Quintiles				.003
Poorest	52 (8.5)	31 (10.4)	21 (6.7)	
Poorer	85 (13.9)	42 (14.1)	43 (13.7)	
Middle	90 (14.7)	58 (19.5)	32 (10.2)	
Richer	156 (25.5)	69 (23.2)	87 (27.6)	
Richest	229 (37.4)	97 (32.7)	132 (41.9)	
Highest Level of Education				.068
No education	53 (8.7)	19 (6.4)	34 (10.8)	
Primary education	175 (28.6)	80 (26.9)	95 (30.2)	
Secondary or higher	384 (62.7)	198 (66.7)	186 (59.0)	
Urban–rural residence				.039
Rural	232 (37.9)	125 (42.1)	107 (34.0)	
Urban	380 (62.1)	172 (57.9)	208 (66.0)	
Province of Residence				< .001
Kinshasa	102 (16.7)	35 (11.8)	67 (21.3)	
Bandundu	79 (12.9)	59 (19.9)	20 (6.3)	
Bas-Congo	70 (11.4)	43 (14.5)	27 (8.6)	
Equateur	53 (8.7)	26 (8.8)	27 (8.6)	
Kasai-Occidental	44 (7.2)	19 (6.4)	25 (7.9)	
Kasai-Oriental	51 (8.3)	26 (8.8)	25 (7.9)	
Katanga	41 (6.7)	27 (9.1)	14 (4.4)	
Maniema	29 (4.7)	13 (4.4)	16 (5.1)	
Nord-Kivu	63 (10.3)	13 (4.4)	50 (15.9)	
Orientale	57 (9.3)	29 (9.8)	28 (8.9)	
Sud-Kivu	23 (3.8)	7 (2.4)	16 (5.1)	
Age				< .001
15–19	31 (5.1)	26 (8.8)	5 (1.6)	
20–24	113 (18.5)	73 (24.6)	40 (12.7)	
25–29	137 (22.4)	78 (26.3)	59 (18.7)	
30–34	138 (22.5)	65 (21.9)	73 (23.2)	
35–39	102 (16.7)	35 (11.8)	67 (21.3)	
40–44	64 (10.5)	16 (5.4)	48 (15.2)	
45–49	27 (4.4)	4 (1.3)	23 (7.3)	
Age at first cohabitation^c^	18.20 (11–38)	18.33 (11–38)	18.08 (11–28)	.391
Currently Working				.572
Yes	446 (73.0)	212 (71.6)	234 (74.3)	
No	165 (27.0)	84 (28.4)	81 (25.7)	
Missing	1			
Religious Affiliation				.567
Christian	582 (95.7)	280 (95.2)	302 (96.2)	
Non-Christian	26 (4.3)	14 (4.8)	12 (3.8)	
Missing	4			
Number of Children				< .001
Less than three	197 (32.2)	130 (43.8)	67 (21.3)	
Three to five	257 (42.0)	118 (39.7)	139 (44.1)	
More than five	158 (25.8)	49 (16.5)	109 (34.6)	

Unweighted descriptive statistic values are reported

^a^Subpopulation of women who reported current use of MC were included in this analysis; Male method of modern contraception = male condom; Female method of modern contraception = female sterilization, IUD, implants/Norplant, injectables, pill, female condom, spermicides, foams, jellies, diaphragm, the morning after pill and the cycle necklace. Women that reported using an “other modern method” were excluded because of the inability to differentiate what type of method was used

^b^P-values from Pearson’s Chi-Square Tests

^c^Mean and range values are reported for age at first cohabitation. P-value from Independent Samples t-test

Table 4 shows the results of the adjusted logistic regression analyses evaluating the association between women’s decision-making power over their own health care and the method of MC used among current users. Bivariate logistic regression showed that women who made decisions about their health care alone had higher odds of using a female method of MC than women whose partner made these decisions alone (OR 1.84; 95% CI 1.01, 3.32). However, after controlling for province of residence, current age, and number of children, the multivariate logistic regression model no longer showed a statistically significant association between women’s independent health care decision-making and type of MC used (OR 1.06; 95% CI 0.58, 1.94). Post hoc analyses showed that women in Nord-Kivu and Sud-Kivu had higher odds of using a female method of MC [(OR 3.82; 95% CI 1.23, 11.87) and (OR 6.07; 95% CI 1.27, 28.93)], respectively, compared with women in Kinshasa. Additionally, women in Bandundu, Bas-Congo, Equateur, and Katanga had lower odds of using a female method of MC [(OR 0.16; 95% CI 0.06, 0.48), (OR 0.22; 95% CI 0.09, 0.54), (OR 0.28; 95% CI 0.10, 0.79) and (OR 0.12; 95% CI 0.04, 0.40)], respectively, compared with women in Kinshasa. In comparison to women aged 15 to 19 years, women in older age groups (30–34; 35–39; 40–44; 45–49) had higher odds of using a female method of MC. Finally, women with three to five children had higher odds of using a female method of MC than women with less than three children (OR 2.01; 95% CI 1.06, 3.82).

**Table 4:** Odds ratios of the method of modern contraception used among current users of modern contraception

	Adjusted OR*^a,b^ [95% CI] (n = 736)
Decision maker for respondent’s health care	
Partner alone	1.00
Respondent and partner	0.81 [0.47, 1.39]
Respondent alone	1.06 [0.58, 1.94]
Province of Residence	
Kinshasa	1.00
Bandundu	**0.16 [0.06, 0.48]**
Bas-Congo	**0.22 [0.09, 0.54]**
Equateur	**0.28 [0.10, 0.79]**
Kasai-Occidental	1.45 [0.42, 4.98]
Kasai-Oriental	0.53 [0.20, 1.43]
Katanga	**0.12 [0.04, 0.40]**
Maniema	2.59 [0.53, 12.61]
Nord-Kivu	**3.82 [1.23, 11.87]**
Orientale	0.60 [0.28, 1.29]
Sud-Kivu	**6.07 [1.27, 28.93]**
Age	
15–19	1.00
20–24	2.95 [0.86, 10.13]
25–29	2.92 [0.94, 9.09]
30–34	**4.22 [1.21, 14.69]**
35–39	**6.01 [1.42, 25.36]**
40–44	**12.67 [3.08, 52.13]**
45–49	**9.28 [1.46, 58.97]**
Number of Children	
Less than three	1.00
Three to five	**2.01 [1.06, 3.82]**
More than five	2.25 [0.92, 5.49]

OR is odds ratio; CI is confidence interval; For the adjusted OR, a complex sample plan was used to weight data and control for sampling method. Women that reported using an “other modern method” were excluded because of the inability to differentiate what type of method was used

Bold = significant at p < 0.05

*Model controls for province of residence, age, and number of children

^a^Subpopulation of women who reported current use of MC were included in this analysis; Male method of modern contraception = male condom; Female method of modern contraception = female sterilization, IUD, implants/Norplant, injectables, pill, female condom, spermicides, foams, jellies, diaphragm, the morning after pill and the cycle necklace

^b^Reference category of the dependent variable is use of a male method of contraception

## Discussion

This study examined the association between Congolese women’s decision-making power over their own health care and current use of MC. Results are consistent with previous findings from Nigeria which suggest that women’s participation in health care decision-making is important in increasing the use of MC [14, 18]. However, contrary to our expectation, women who made health care decisions alone, as opposed to those who made decisions jointly with their husband/partner, were more likely to use MC. This contradicts the previous finding that relational autonomy, as determined by joint participation in health care decisions, is important in increasing the use of MC [19, 20].

While not the primary focus of our study, we observed that women who participated jointly with their partner in decisions about how their partner’s earnings were used were more likely to use MC compared to women who had no say. Since a large proportion of the health system in the DRC is financed by out-of-pocket expenses, jointly deciding to invest in contraception may be important [32, 33]. Additionally, the role of financial decision-making in contraceptive use has likely become more important in recent years due to increasing interest in the use of contraceptive implants. A study in Kinshasa found that from 2013 to 2017, the proportion of MC users with long-acting reversible contraception increased from 10.8% to 40.0% [7, 8]. This was almost exclusively due to increased use of the implant [7, 8]. However, with cost being reported as a major barrier to Congolese women using contraceptive implants [11], joint decision-making about contraception may be more important from a financial rather than a health care standpoint.

In addition to decision-making, there were other variables that were statistically significantly associated with current use of MC in this study population. For example, women with secondary or higher levels of education were more likely to use MC than women with no education. This relationship between education level and use of MC is consistent with previous findings and supports the idea that women with higher levels of education have a better understanding of health and are more assertive about their needs [13–15, 20]. Additionally, consistent with previous literature, wealth was found to be an important determinant of MC use [13–15]. This is likely because Congolese individuals are often required to pay out-of-pocket for contraception, which, as described above, is likely a major barrier to contraceptive use given that a high proportion of the population lives below the poverty line [11, 32, 33]. Moreover, women in urban areas were more likely to use MC than women in rural regions, which may suggest that women in urban areas have better access to reproductive health care services.

In 1981, the Primary Health Care (PHC) strategy was enacted in the DRC, which states that, among other services, family planning must be available at primary healthcare centers [6]. Furthermore, in 2008, the National Policy for Reproductive Health (RH) was revised to normalize family planning services and make them accessible in rural and urban regions [6]. However, implementation of these services at health facilities has been challenging [6]. Family planning arguably only came into the government’s agenda in a meaningful way in 2012 when the Permanent Multisectoral Technical Committee (CTMP) began developing a National Strategic Plan for Family Planning, which was released in February 2014 [34]. Additionally, at the 2013 International Conference on Family Planning, the DRC was confirmed as a member of the global Family Planning 2020 (FP2020) partnership to increase MC access for women and girls [35]. This also brought light to the funds that were allotted by the government to MC in 2013 [34]. However, these funds are not a fixed budget item for the Ministry of Health, and turnover in the government means that commitment to funding family planning is not guaranteed for upcoming years [34]. Therefore, in order to ensure the availability and accessibility of MC across the DRC, there needs to be continued financial contributions from the government and improvements in service delivery through the coordination of various family planning organizations [33, 34].

Despite these efforts, there are still numerous sociocultural barriers to contraceptive use, particularly for women. Out of 160 countries, the DRC ranks 152 in the Gender Inequality Index and this inequality is exemplified in the limited decision-making control that many women have [22, 36]. Additionally, a study in Kinshasa found that men often feel that contraception cannot be used because God creates a natural order and this must be respected [11]. A study in rural DRC also found that many individuals feel that their community idealizes having many children as children are God’s gift [12]. These religious factors, as well as cultural factors, may lead to communities viewing family planning unfavourably [12]. Other common reasons for having a negative attitude towards MC use include beliefs that it promotes prostitution and causes sterility [12]. Awareness raising to overcome misperceptions about MC and gender sensitization are both needed to increase contraception uptake at the community level. Activities to increase MC use and improve gender equality will contribute to Sustainable Development Goals (SDG) 5.6 and 3.1 by helping to increase access to sexual and reproductive health and rights and reduce maternal mortality [16].

Finally, among current users of MC, no association was found between health care decision-making power and method of MC used. To our knowledge, the only other published study in sub-Saharan Africa to investigate the relationship between health care decision-making power and method of MC used found that Zambian women who made health care decisions jointly with their partner were more likely to use long-acting and permanent contraceptive methods than Zambian women who did not make joint decisions [21]. This suggests that the permanence of the method may also be important to consider. Further studies should investigate the relationship between women’s decision-making power and method of MC used in a more granular way.

Interestingly, other variables, such as province of residence, were significantly associated with the method of MC used in the current study. For example, women in Nord-Kivu and Sud-Kivu were significantly more likely to use female methods of MC than women in Kinshasa. These two provinces are located in eastern DRC which has been affected by armed conflict over the last two decades [9]. Therefore, higher rates of female modern contraceptive use may be a result of the concentration of humanitarian aid in these areas [37]. This is particularly likely given that aid organizations may aim to provide a range of contraceptive methods, including IUDs and implants, which are highly utilized [9, 38]. Additionally, older women were found to be more likely to use female methods of MC than younger women. This may be because some female methods of contraception, such as the IUD and implant, are long-acting. Long-acting methods may be preferred by older women because their need to space or limit births may be greater.

## Strengths and limitations

A major strength of this study was that the data were collected at the population level and therefore are more generalizable in the DRC. Additionally, there was a high response rate, with 99% of women agreeing to partake in the survey [2]. High response rates in DHS surveys have been attributed to the thorough training of staff and supervisors, the short time frame between obtaining household lists and conducting the survey, and the high levels of cooperation commonly seen in developing countries as opposed to developed countries [39]. The survey also contained a variety of questions on demographic, health and social factors, which allowed for adequate assessment of known confounders [2]. However, the study has some notable limitations. Firstly, the cross-sectional nature of the study does not allow for determination of a temporal relationship between health care decision-making power and use of MC. Nevertheless, it is unlikely that the use of contraception influenced women’s decision-making power over their own health care [40]. Secondly, there is the possibility of measurement error in the exposure variable because of the crude nature of the question asked in the survey questionnaire. In patriarchal societies, women may report that a joint decision was made even when they were forced to agree with the decision of their partner [41]. Thirdly, women in the first six weeks postpartum are not in need of contraception, but they were unable to be selectively removed from the analysis [42]. However, due to the large size of the study population, these women are unlikely to have significantly biased the results. Fourthly, there is the possibility of interviewer bias and social desirability bias given that sensitive questions were asked in face-to-face interviews. However, local interviewers were extensively trained and measures were taken to ensure that women answered questions privately, which likely minimized the effects of these biases [2]. Finally, while it is recognized that the 2013–14 DHS data may not represent present day conditions, it is the most recently conducted household survey in DRC.

## Conclusions

This study adds to the body of literature investigating the relationship between women’s decision-making power and use of MC in the DRC. Specifically, our results suggest that women who make decisions about their health care alone are more likely to use MC. Additionally, we observed that women who engage in joint decision-making with their partner about their partner’s earnings may be more likely to use MC. These findings have two main implications for programs aimed at increasing the use of MC in the DRC. Firstly, interventions promoting women’s autonomy and right to independently make decisions regarding their own bodies may aid in increasing use of MC. This is also important in working towards the SDGs of good health and gender equality [16, 20]. Secondly, programs should consider implementing interventions aimed at improving the ability of Congolese women to negotiate with their partner about how his earnings are spent. Further research is needed to qualitatively assess the relationship between different domains of decision-making in relation to MC use. Specifically, given the societal and cultural barriers to MC use, qualitative research is needed to understand the perspectives of men, women and couples on how decisions regarding MC are, or should be, made. Doing so may provide additional guidance on how to most effectively deliver interventions that enable women to negotiate for the use of MC.

## Data Availability

The dataset analysed during the current study is available in the DHS repository: https://dhsprogram.com/data/available-datasets.cfm.

## Abbreviations

DRC: Democratic Republic of the Congo
DRC-DHS: DRC Demographic and Health Survey
CI: Confidence Interval
IUD: Intrauterine Device
MC: Modern Contraception
OR: Odds Ratio
SDG: Sustainable Development Goals

## References

[1] Cleland J, Conde-Agudelo A, Peterson H, Ross J, Tsui A (2012). Contraception and health. Lancet.

[2] Ministère du Plan et Suivi de la Mise en œuvre de la Révolution de la Modernité (MPSMRM), Ministère de la Santé Publique (MSP), ICF International. Enquête Démographique et de Santé en République Démocratique du Congo 2013–2014. Rockville, Maryland, USA; 2014. https://dhsprogram.com/pubs/pdf/FR300/FR300.pdf.

[3] Ministère du Plan, Macro International. Enquête Démographique et de Santé, République Démocratique du Congo 2007. Calverton, Maryland, USA; 2008. https://dhsprogram.com/pubs/pdf/FR208/FR208.pdf.

[4] Ghana Statistical Service, Ghana Health Service, ICF International. Ghana Demographic and Health Survey. Rockville, Maryland. USA. 2014;2015. 10.15171/ijhpm.2016.42.

[5] Kenya National Bureau of Statistics, Ministry of Health/Kenya, National AIDS Control Council, Kenya Medical Research Institute, National Council for Population Development, ICF International. Kenya Demographic and Health Survey 2014. Rockville, MD, USA; 2014. https://dhsprogram.com/pubs/pdf/FR308/FR308.pdf.

[6] Democratic Republic of the Congo Ministry of Public Health. Family Planning: National Multisectoral Strategic Plan (2014–2020). 2014. http://advancefamilyplanning.org/sites/default/files/resources/PSN_PF_2014-2020_english_20140916%27.pdf.

[7] Performance Monitoring and Accountability 2020. PMA2020/Kinshasa, DRC: September-November 2017 (Round 6). 2017. https://pma2020.org/sites/default/files/PMA2020-Kinshasa-DRC-R6-FP-Brief-En.pdf.

[8] Performance Monitoring and Accountability 2020. PMA2013/Kinshaha-R1. 2017. https://pma2020.org/sites/default/files/DRC-Kinshasa-R1-EN-FP-Brief-v8-2017-07-13_0.pdf.

[9] Casey SE, Tshipamba M (2017). Contraceptive availability leads to increase in use in conflict-affected Democratic Republic of the Congo: Evidence from cross-sectional cluster surveys, facility assessments and service statistics. Confl Health.

[10] Casey SE, Gallagher MC, Dumas EF, Kakesa J, Katsongo JM, Muselemu J-B (2019). Meeting the demand of women affected by ongoing crisis: Increasing contraceptive prevalence in North and South Kivu, Democratic Republic of the Congo. PLoS ONE.

[11] Muanda M, Ndongo PG, Taub LD, Bertrand JT (2016). Barriers to modern contraceptive use in Kinshasa. DRC PLoS One.

[12] Muanda MF, Ndongo GP, Messina LJ, Bertrand JT (2017). Barriers to modern contraceptive use in rural areas in DRC. Cult Health Sex.

[13] Sano Y, Antabe R, Atuoye KN, Braimah JA, Galaa SZ, Luginaah I (2018). Married women’s autonomy and post-delivery modern contraceptive use in the Democratic Republic of Congo. BMC Womens Health.

[14] Lamidi EO (2015). State variations in women’s socioeconomic status and use of modern contraceptives in Nigeria. PLoS ONE.

[15] Wado YD (2017). Women’s autonomy and reproductive health-care-seeking behavior in Ethiopia. Women Health.

[16] United Nations. Transforming our world: The 2030 agenda for sustainable development. 2015. https://sustainabledevelopment.un.org/index.php?page=view&type=400&nr=2125&menu=1515.

[17] OlaOlorun FM, Hindin MJ (2014). Having a say matters: Influence of decision-making power on contraceptive use among Nigerian women ages 35–49 years. PLoS ONE.

[18] Asaolu IO, Okafor CT, Ehiri JC, Dreifuss HM, Ehiri JE (2017). Association between measures of women’s empowerment and use of modern contraceptives: an analysis of Nigeria’s Demographic and Health Surveys. Front Public Heal.

[19] Islam AZ (2018). Factors affecting modern contraceptive use among fecund young women in Bangladesh: Does couples’ joint participation in household decision making matter?. Reprod Health.

[20] Osamor P, Grady C (2016). Women’s autonomy in health care decision-making in developing countries: A synthesis of the literature. Int J Womens Health.

[21] Mutombo N, Bakibinga P (2014). The effect of joint contraceptive decisions on the use of Injectables, Long-Acting and Permanent Methods (ILAPMs) among married female (15–49) contraceptive users in Zambia: a cross-sectional study. Reprod Health.

[22] Ministère du Plan et Suivi de la Mise en œuvre de la Révolution de la Modernité (MPSMRM), Ministère de la Santé Publique (MSP), ICF International. Democratic Republic of Congo Demographic and Health Survey 2013–14: Key Findings. Rockville, Maryland, USA; 2014. https://dhsprogram.com/pubs/pdf/SR218/SR218.e.pdf.

[23] Chirwa TF, Mantempa JN, Kinziunga FL, Kandala JD, Kandala N-B (2014). An exploratory spatial analysis of geographical inequalities of birth intervals among young women in the Democratic Republic of Congo (DRC): A cross-sectional study. BMC Pregnancy Childbirth.

[24] Kandala N-B, Lukumu FK, Mantempa JN, Kandala JD, Chirwa T (2015). Disparities in modern contraception use among women in the Democratic Republic of Congo: a cross-sectional spatial analysis of provincial variations based on household survey data. J Biosoc Sci.

[25] Uddin J, Hossin MZ, Pulok MH (2017). Couple’s concordance and discordance in household decision-making and married women’s use of modern contraceptives in Bangladesh. BMC Womens Health.

[26] Ranganathan P, Pramesh CS, Aggarwal R. Common pitfalls in statistical analysis: Logistic regression. Perspect Clin Res. 2017;8:148–51. https://www.ncbi.nlm.nih.gov/pmc/articles/PMC5543767/. 10.4103/picr.PICR_87_17 PMC5543767 28828311

[27] Budtz-Jørgensen E, Keiding N, Grandjean P, Weihe P (2007). Confounder selection in environmental epidemiology: assessment of health effects of prenatal mercury exposure. Ann Epidemiol.

[28] Greenland S, Daniel R, Pearce N (2016). Outcome modelling strategies in epidemiology: Traditional methods and basic alternatives. Int J Epidemiol.

[29] Midi H, Sarkar SK, Rana S (2010). Collinearity diagnostics of binary logistic regression model. J Interdiscip Math.

[30] Chan YH. Biostatistics 202: Logistic regression analysis. Singapore Med J. 2004;45:149–53. https://www.sma.org.sg/smj/4504/4504bs1.pdf. 15094982

[31] von Elm E, Altman DG, Egger M, Pocock SJ, Gøtzsche PC, Vandenbroucke JP (2007). Strengthening the reporting of observational studies in epidemiology (STROBE) statement: guidelines for reporting observational studies. BMJ.

[32] Barroy H, Andre F, Mayaka S, Samaha H. Investing in universal health coverage: Opportunities and challanges for health financing in the Democratic Republic of Congo. 2014. http://documents.worldbank.org/curated/en/782781468196751651/pdf/103444-WP-P147553-PUBLIC-Health-PER-Investing-in-Universal-Health-1608488.pdf.

[33] Kwete D, Binanga A, Mukaba T, Nemuandjare T, Mbadu MF, Kyungu M-T (2018). Family planning in the Democratic Republic of the Congo: Encouraging momentum, formidable challenges. Glob Heal Sci Pract.

[34] Mukaba T, Binanga A, Fohl S, Bertrand JT (2015). Family planning policy environment in the Democratic Republic of the Congo: levers of positive change and prospects for sustainability. Glob Heal Sci Pract.

[35] Advance Family Planning. Democratic Republic of the Congo commits to family planning through the global FP2020 partnership. 2015. http://advancefamilyplanning.org/sites/default/files/resources/drc_EN.pdf.

[36] United Nations Development Fund. Human development indices and indicators: 2018 statistical update - Congo (Democratic Republic of the). 2018. http://hdr.undp.org/sites/all/themes/hdr_theme/country-notes/COD.pdf.

[37] Kidman R, Palermo T, Bertrand J (2015). Intimate partner violence, modern contraceptive use and conflict in the Democratic Republic of the Congo. Soc Sci Med.

[38] Rattan J, Noznesky E, Curry DW, Galavotti C, Hwang S, Rodriguez M (2016). Rapid contraceptive uptake and changing method mix with high use of long-acting reversible contraceptives in crisis-affected populations in Chad and the Democratic Republic of the Congo. Glob Heal Sci Pract.

[39] Vaessen M, Thiam M, Le T. The Demographic and Health Surveys. In: Household Sample Surveys in Developing and Transition Countries. 2005. p. 495–503. https://unstats.un.org/unsd/hhsurveys/pdf/household_surveys.pdf.

[40] AlSumri HH. A national study: The effect of Egyptian married women’s decision-making autonomy on the use of modern family planning methods. Afr J Reprod Health. 2015;19:68–77. https://www.ncbi.nlm.nih.gov/pubmed/27337855. 27337855

[41] Senarath U, Gunawardena NS (2009). Women’s autonomy in decision making for health care in South Asia. Asia Pacific J Public Heal.

[42] World Health Organization. Programming strategies for postpartum family planning. 2013. https://apps.who.int/iris/bitstream/handle/10665/93680/9789241506496_eng.pdf?sequence=1.

